# Comparative review of pharmacological therapies in individuals with HER2-positive advanced breast cancer with focus on hormone receptor subgroups

**DOI:** 10.3389/fonc.2022.943154

**Published:** 2022-08-18

**Authors:** Chinyereugo M. Umemneku-Chikere, Olubukola Ayodele, Marta Soares, Sam Khan, Keith Abrams, Rhiannon Owen, Sylwia Bujkiewicz

**Affiliations:** ^1^ Biostatistics Research Group, Department of Health Sciences, University of Leicester, Leicester, United Kingdom; ^2^ University Hospital Leicester National Health Service (NHS) Trust, Leicester Royal Infirmary, Leicester, United Kingdom; ^3^ Centre for Health Economics, University of York, York, United Kingdom; ^4^ Leicester Cancer Research Centre, University of Leicester, Leicester, United Kingdom; ^5^ Department of Statistics, University of Warwick, Coventry, United Kingdom; ^6^ Medical School, Swansea University, Swansea, United Kingdom

**Keywords:** Advanced breast cancer, hormone receptor, HER2 positive, metastatic breast cancer, targeted therapies, network meta-analysis, subgroup analysis

## Abstract

Breast cancer is the fifth leading cause of cancer-related deaths worldwide. The randomized controlled trials (RCTs) of targeted therapies in human epidermal receptor 2 (HER2)–positive advanced breast cancer (ABC) have provided an evidence base for regulatory and reimbursement agencies to appraise the use of cancer therapies in clinical practice. However, a subset of these patients harbor additional biomarkers, for example, a positive hormone receptor status that may be more amenable to therapy and improve overall survival (OS). This review seeks to explore the reporting of evidence for treatment effects by the hormone receptor status using the RCT evidence of targeted therapies for HER2-positive ABC patients. Preferred Reporting Items for Systematic Reviews and Meta-Analysis (PRISMA) guidelines were followed to identify published RCTs. Extracted data were synthesized using network meta-analysis to obtain the relative effects of HER2-positive-targeted therapies. We identified a gap in the reporting of the effectiveness of therapies by the hormone receptor status as only 15 out of 42 identified RCTs reported hormone receptor subgroup analyses; the majority of which reported progression-free survival but not OS or the overall response rate. In conclusion, we recommend that future trials in ABC should report the effect of cancer therapies in hormone receptor subgroups for all outcomes.

## Introduction

Breast cancer is the most commonly diagnosed cancer and the fifth leading cause of cancer-related deaths worldwide (1). Advances in breast cancer screening and radiological and surgical techniques have helped to improve overall survival (OS) rates. Additionally, a deeper understanding of the underlying molecular drivers of breast cancer pathogenesis has led to the development of a range of targeted treatments, for example, to hormone receptors, human epidermal receptor 2 (HER2) receptors, or programmed death receptor ligand 1, allowing an era of personalized medicine to be realized (2). When considering HER2-positive breast cancer, the examples of targeted therapies include trastuzumab, lapatinib, trastuzumab emtansine, trastuzumab deruxtecan, and neratinib (3). The efficacy of these therapies has been demonstrated in randomized controlled trials (RCTs), leading to their market access approval by regulatory agencies, such as the European Medicines Agency and Food and Drug Administration in the US. These have been subsequently appraised by reimbursement agencies such as the National Institute for Health and Care Excellence (NICE) in the UK for use in routine clinical practice. The NICE determines clinical and cost-effectiveness (or value for money) for the population covered in the full market authorization. However, they may consider the use of subgroups (such as subgroups defined by the hormone-receptor biomarker status) if evidence shows an unclear value for money within one of the groups or in subgroups where patients are known to have improved prognosis. For example, the NICE appraisal of lapatinib or trastuzumab in combination with an aromatase inhibitor (AI) is recommended as the first-line treatment of HER2-positive advanced breast cancer (ABC), in the hormone-receptor-positive (HR+ve) population only (TA257; https://www.nice.org.uk/guidance/ta257). This review was undertaken to ascertain if there is available RCT evidence on the hormone-receptor status in HER2-positive ABC, as to whether the hormone-receptor status has a bearing on the clinical outcomes of individuals being treated for HER2-positive ABC. Specifically, we investigated the level of reporting of RCT results by the hormone-receptor status and explore whether the effectiveness of therapies in HER2-positive ABC patients varies according to the hormone-receptor status (i.e., estrogen and or progesterone biomarker status). Hormone-receptor subgroups were established as the HR+ve subgroup, which includes patients with a positive estrogen and/or progesterone receptor status, and the hormone-receptor-negative (HR-ve) subgroup, which includes patients whose status for both estrogen and progesterone were negative. Evidence from the identified trials was synthesized to estimate the effect of treatments on progression-free survival (PFS) in HR+ve or HR-ve subgroups. The next section in this paper discusses the methods used in this review, the results are discussed in section three, and section four concludes with a summary of the findings, recommendations, limitations, and further research.

## Methodology

### Literature review

RCTs were identified following a systematic approach, with a review of reviews carried out first followed by a search of more recent RCTs. The first step identified all the trials used as evidence in technology appraisals by the NICE for targeted therapies in HER2-positive ABC patients. This was followed by identifying reviews, systematic reviews, meta-analysis, and network meta-analysis published in peer-reviewed journals that included the RCTs of women with HER2-positive ABC (4–23, 25–29). This approach was employed to utilize comprehensive systematic reviews and network meta-analyses that included the RCTs of targeted therapies for HER2-positive ABC patients. The final step was an additional search for more recent RCTs evaluating targeted therapies among HER2-positive ABC patients. The eligibility criteria for the selection of RCTs and search terms are listed below.

### Eligible criteria of selecting randomized controlled trials

The eligibility of the RCTs for inclusion in this study was defined by the following criteria for the population, interventions, comparators, and outcomes (PICOs):

- Phase two and three RCTs focusing primarily on female patients with HER2-positive ABC
- All treatments (interventions and comparators) targeted at HER2-positive ABC
- RCTs that reported at least one of the following outcomes: OS, PFS, and overall response rate (ORR)

RCTs excluded were:

- Studies reporting only outcomes with adverse effect or patients
- Studies focusing on treatment dose escalation and the biosimilar studies of trastuzumab
- Single-arm studies
- Studies involving only postmenopausal women, patients with brain metastasis, leptomeningeal meningitis, or central nervous system metastases to ensure the homogeneity of the trial populations across treatments

### Search strategies

**Figure 1:**
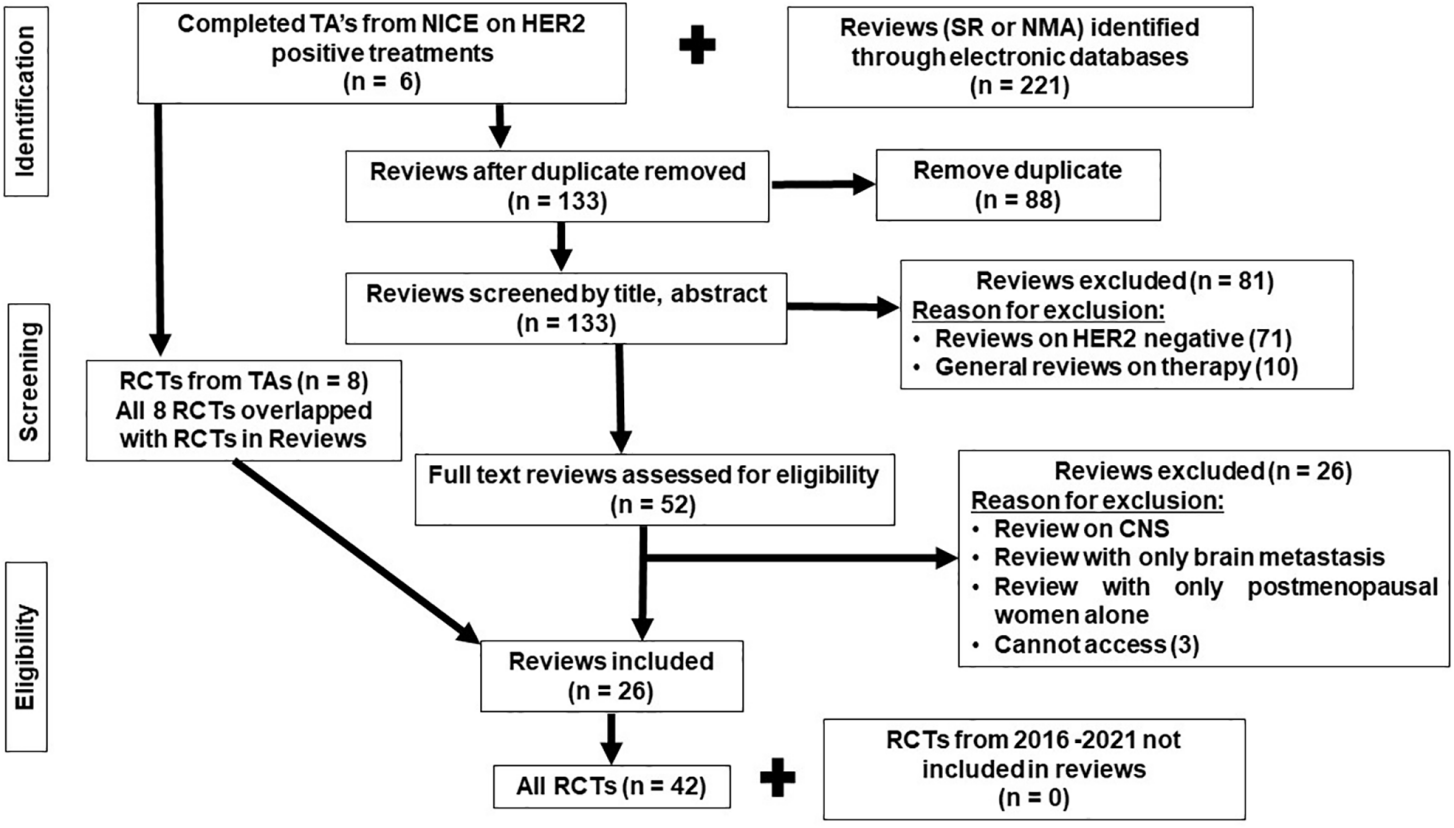
PRISMA flowchart of randomized controlled trials (RCTs) included in the review.

The search of the systematic reviews covered NICE guidelines, PubMed, Cochrane Library, and Scopus, with the search covering the period from the inception of the databases to 20 March 2022. More recent RCTs were then searched for within Scopus and PubMed, published in the last 6 years (2016–2022) to ensure that more recent RCTs were included. The PRISMA flow chart presenting all stages of study selection is shown in **Figure 1**. The search terms are included in the **Supplementary File 1**.

### Statistical methods

Network meta-analyses (NMAs) were carried out to assess the efficacy of treatments identified in the review. Firstly, NMA was conducted using all the identified RCTs that formed a connected network (i.e., the trial had at least one treatment arm in common with another trial in the network) irrespective of whether the trial reported subgroups analyses or not. Secondly, NMA was conducted using information reported for hormone-receptor subgroups. The experimental treatments and comparators of the identified RCTs included in the NMAs are different, and thus, in order to make comparisons across treatments, a reference treatment comparator needed to be identified. The reference treatment comparator was selected as the most commonly evaluated treatment in the connected networks, or where there were multiple common treatment comparisons; then, the most efficacious treatment was selected (30). The efficacy of the treatments in the network including all HER2-positive patients were assessed based on PFS, OS, and the ORR. Treatment effects on PFS and OS were measured using hazard ratios (HRs), and the effects on ORR were measured using odds ratios (ORs). The comparative efficacy of cancer therapies by hormone-receptor subgroups was based on PFS, which was the most commonly reported outcome in the identified RCTs. A random-effects (31, 32) NMA in a Bayesian framework was used to synthesize evidence from the identified trials. The analyses were performed using the WinBUGS 1.4.3 software. The effectiveness estimates were reported as means and corresponding 95% credible intervals (Crls). Non-informative prior distributions were used with the full WinBUGS code provided in the Technical Support Document (33).

## Results

### All randomized controlled trial network results

Forty-two published RCTs focusing on treatments administered to HER2-positive ABC patients were identified from 26 reviews and four NICE technology appraisals (TAs) (34–80). The eight RCTs identified from the TAs overlapped with the RCTs identified in the reviews. There were no additional RCTs identified from the additional search (of RCTs published between 2006 and 2022) that have not been included in the reviews (**Figure 1**). All RCTs meeting the eligibility criteria and included in the review were phase II and phase III.

**Figure 2:**
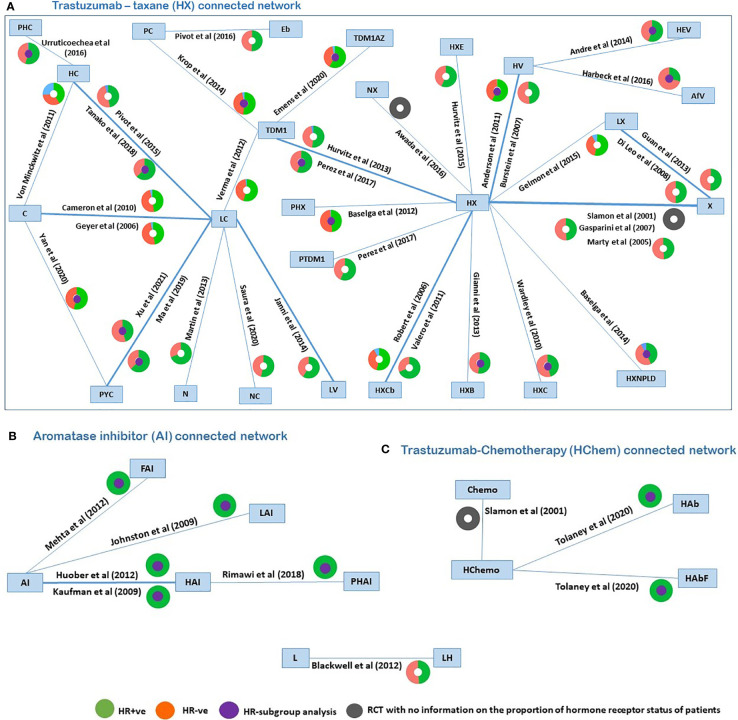
Network plots of identified trials (reporting PFS), with colors in the circles representing the proportion of patients in each RCT that are HR+ve (orange), HR-ve (green), unknown (blue), not reported (grey), and the middle purple circle indicated RCTs reporting subgroup analyses. PHC, pertuzumab + trastuzumab + capecitabine; PC, physician choice; LC, lapatinib + capecitabine; TDM1, trastuzumab emtansine; C, capecitabine; PYC, pyrotinib + capecitabine; LV, lapatinib + vinorelbine; HC, trastuzumab + capecitabine; N, neratinib; TDM1AZ, trastuzumab emtansine + atezolizumab; NX, neratinib + taxane; X, taxane (paclitaxel or docetaxel);NC, neratinib + capecitabine; HX, trastuzumab + taxane; HXB, trastuzumab + taxane + bevacizumab; LX, lapatinib + taxane; HV, trastuzumab + vinorelbine; HXE, trastuzumab + taxane + everolimus; PHX, pertuzumab + trastuzumab + taxane; HXC, trastuzumab + capecitabine + taxane; AfV, afatinib + vinorelbine; HEV, trastuzumab + everolimus + vinorelbine; HXCb, trastuzumab + taxane + carboplatin; PTDM1, pertuzumab + trastuzumab emtansine; Chemo, standard chemotherapy; LH, lapatinib + trastuzumab; L, lapatinib; AI, aromatase inhibitors (letrozole or anastrozole); LAI, lapatinib + AI; FAI, fulverstrant + AI; HAI, trastuzumab +AI; PHAI, pertuzumab + trastuzumab +AI; HAb, trastuzumab + abemaciclib; HAbF, trastuzumab + abemaciclib + fulverstrant; HXNPLD, trastuzumab + taxane + NPLD; NPLD, non-pegylated liposomal doxorubicin; HChemo, trastuzumab + chemotherapy; Eb, eribulin.

A network diagram of all 42 trials (reporting PFS) is displayed in **Figure 2**, similarly as in Cope et al. (81). **Figure 2** included three networks of trials (with at least one arm common with another trial, thus forming a network) disconnected from each other due to a lack of a common comparator. In the plot (**Figure 2**), different colors in the circles indicate the proportion of patients in each RCT that are HR+ve (orange), HR-ve (green), unknown (blue), and not reported (gray). The trials reporting subgroup analyses by the hormone-receptor status are highlighted with a purple circle in the middle of a colored circle. Six RCTs recruited HR+ve patients, and of the 36 RCTs recruiting the mixed populations of HR+ve and HR-ve patients, only 15 RCTs reported separate hormone receptor subgroup analyses. The identified RCTs do not all form a connected network for the broader population; hence, three connected networks were investigated. These connected networks are the trastuzumab–taxane (HX)–connected network (**Figure 2A**), AI-connected network (**Figure 2B**), and the trastuzumab–chemotherapy (HChem)–connected network (**Figure 2C**). Paclitaxel and docetaxel, which inhibit microtubule dynamics, were classified as a taxane. Letrozole and anastrozole, which are non-steroid third-generational AIs that interfere with the production of estrogen, were classified as AIs (30, 82–85). NMAs were carried out to compare treatments that form each of the smaller connected networks. A list of all included RCTs is provided in the **Supplementary File 2**.

**Figure 3:**
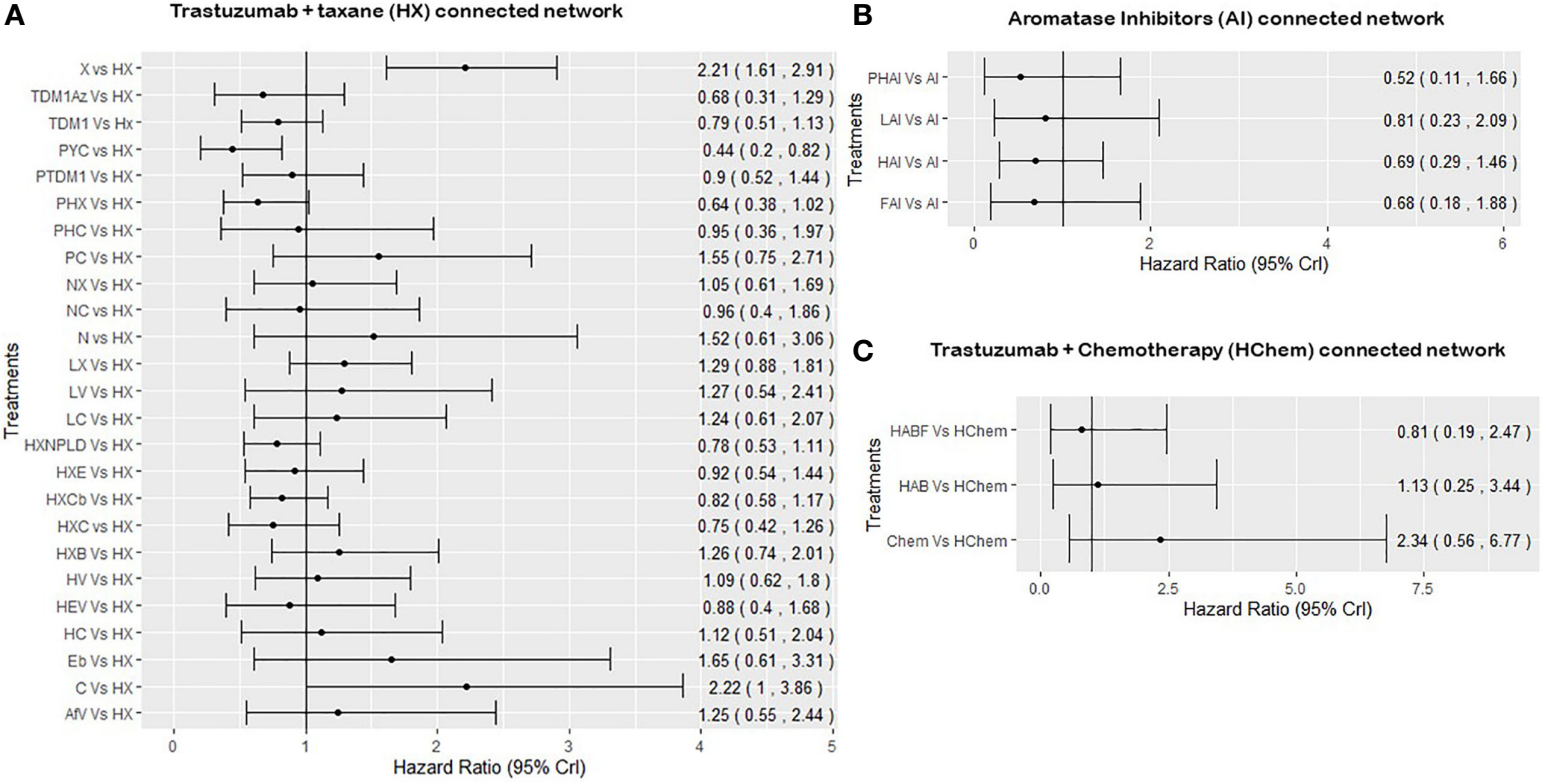
Summary forest plots obtained from the NMA including all RCTs for PFS. PHC, pertuzumab + trastuzumab + capecitabine; PC, physician choice; LC, lapatinib + capecitabine; TDM1, trastuzumab emtansine; C, capecitabine; PYC, pyrotinib + capecitabine; LV, lapatinib + vinorelbine; HC, trastuzumab + capecitabine; N, neratinib; TDM1AZ, trastuzumab emtansine + atezolizumab; NX, neratinib + taxane; X, taxane (paclitaxel or docetaxel);NC, neratinib + capecitabine; HX, trastuzumab + taxane; HXB, trastuzumab + taxane + bevacizumab; LX, lapatinib + taxane; HV, trastuzumab + vinorelbine; HXE, trastuzumab + taxane + everolimus; PHX, pertuzumab + trastuzumab + taxane; HXC, trastuzumab + capecitabine + taxane; AfV, afatinib + vinorelbine; HEV, trastuzumab + everolimus + vinorelbine; HXCb, trastuzumab + taxane + carboplatin; PTDM1, pertuzumab + trastuzumab emtansine; Chemo, standard chemotherapy; LH, lapatinib + trastuzumab; L, lapatinib; AI, aromatase inhibitors (letrozole or anastrozole); LAI, lapatinib + AI; FAI, fulverstrant + AI; HAI, trastuzumab +AI; PHAI, pertuzumab + trastuzumab +AI; HAb, trastuzumab + abemaciclib; HAbF, trastuzumab + abemaciclib + fulverstrant; HXNPLD, trastuzumab + taxane + NPLD; NPLD, non-pegylated liposomal doxorubicin; HChemo, trastuzumab + chemotherapy; Eb, eribulin. Treatment effects are considered to be statistically significance if the 95% credible interval does not include the point of no difference which, 1.

For the network of treatment comparisons for the total population (**Figure 2**), HX was the most commonly evaluated intervention and was thus used as the reference treatment comparator. The treatment effect estimates and corresponding 95% Crls for PFS in this population for each connected network are provided in **Figure 3**. In the overall NMA, taxane showed an important increase in the risk of disease progression compared to HX with a hazard ratio of 2.21 (95% Crl: 1.61, 2.91); pyrotinib + capecitabine (PYC) showed an important reduction in the risk of progression compared to HX with a hazard ratio of 0.44 (0.20, 0.82); and capecitabine appeared to show a meaningful increase in the risk of progression compared to HX with a hazard ratio of 2.22 (1.00, 3.86). Other treatments evaluated using HX as the reference treatment did not show a meaningful difference in effect as their 95% CrI spans the point of no difference (1). The relative treatment effects (for all treatment comparisons in the network) for PFS, OS, and ORR are reported in the **Supplementary File 3**. For example, HER2-positive-targeted therapies combined with taxane—such as lapatinib with taxane (LX), neratinib with taxane (NX), trastuzumab with taxane and bevacizumab (HXB), trastuzumab with taxane and carboplatin (HXCb), trastuzumab with taxane and capecitabine (HC), trastuzumab with taxane and pertuzumab (PHX), trastuzumab with everolimus and taxane (HXE), and trastuzumab with taxane and non-pegylated liposomal doxorubicin (HXNPLD)—and some targeted therapies like trastuzumab emtansine (TDM1) and neratinib with capecitabine all had an important decreased risk of disease progression compared to taxane alone. In addition, TDM1 (using the point estimates) showed to prolong overall survival when compared to other HER2-positive-targeted therapies like HX, HC, LC, taxane, and LX (see **Supplementary File 3**). Pertuzumab with TDM1 (PTDM1) showed a meaningful decreased risk in disease progression compared to LC, capecitabine, taxane, and neratinib. The relative treatment effects of all treatments evaluated in the mixed and hormone receptor subgroup population are reported in the **Supplementary File 3**. PYC showed a meaningful decreased risk in disease progression compared to some targeted therapies such as HX, TDM1, LX, and trastuzumab with capecitabine. The meaningful treatment effects showed by PYC could be associated with the fact that pyrotinib is an irreversible inhibitor of the ERBB family including HER1, HER2, and HER4; therefore, potentially allowing wider HER2 inhibition compared to other anti-HER2 therapies. In addition, PYC was evaluated only as a second line of therapy, which may have had an impact on the results from the NMA as we discuss in more detail in the Discussion section. For the AI-connected network (**Figure 2B**), only HR+ve patients were included as the AI therapies are only used in the HR+ve breast cancer setting (84).

### Results of subgroup analyses

**Figure 4:**
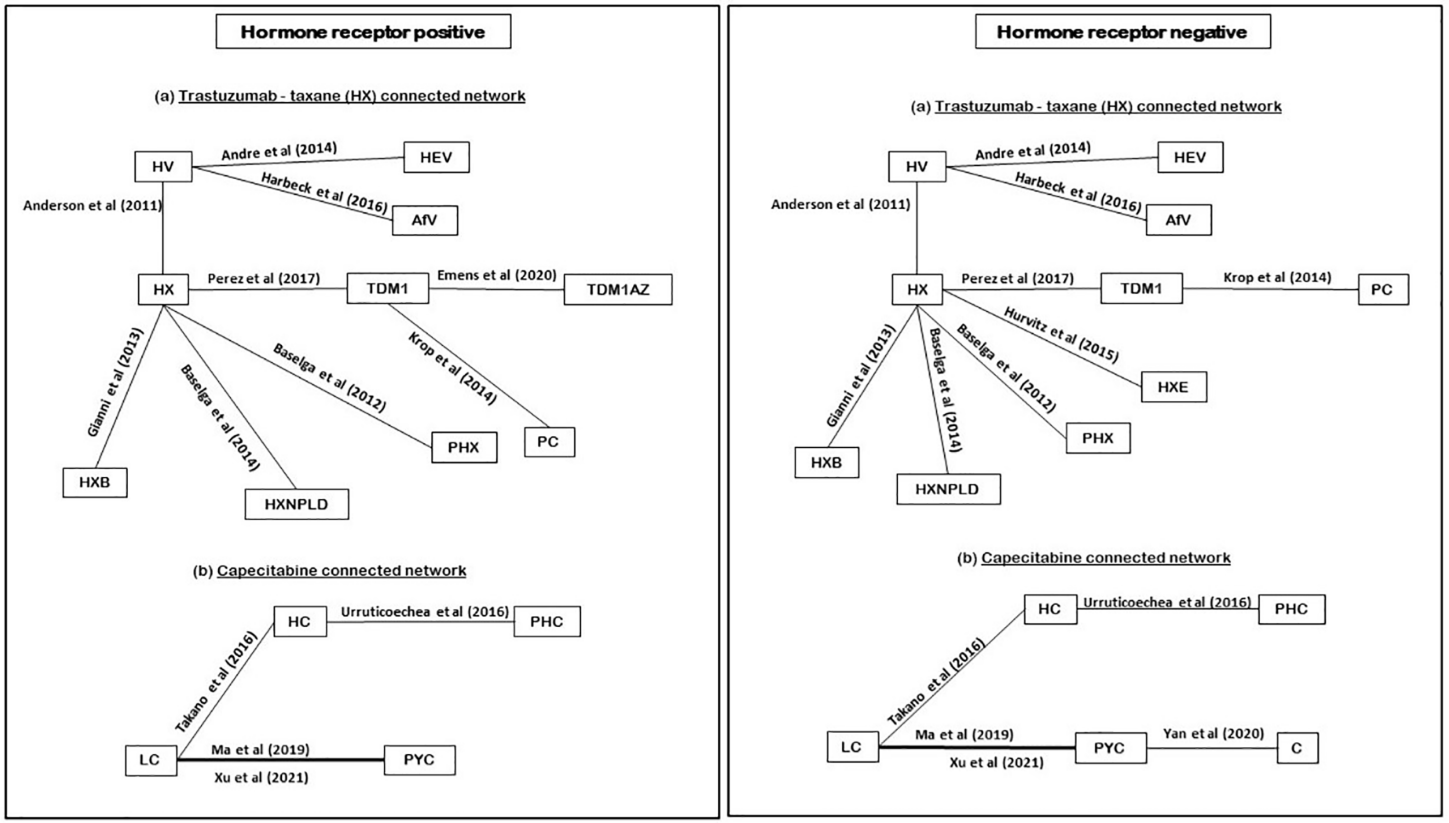
Network plot of hormone receptors subgroup RCTs (reporting PFS). PHC, pertuzumab + trastuzumab + capecitabine; PC, physician choice; LC, lapatinib + capecitabine; TDM1, trastuzumab emtansine; C, capecitabine; PYC, pyrotinib + capecitabine; HC, trastuzumab + capecitabine; TDM1AZ, trastuzumab emtansine + atezolizumab; HX, trastuzumab + taxane; HXB, trastuzumab + taxane + bevacizumab; LX, lapatinib + taxane; HV, trastuzumab + vinorelbine; HXE, trastuzumab + taxane + everolimus; PHX, pertuzumab + trastuzumab + taxane; AfV, afatinib + vinorelbine; HEV, trastuzumab + everolimus + vinorelbine; HXNPLD, trastuzumab + taxane + NPLD; NPLD, non-pegylated liposomal doxorubicin.

**Figure 5:**
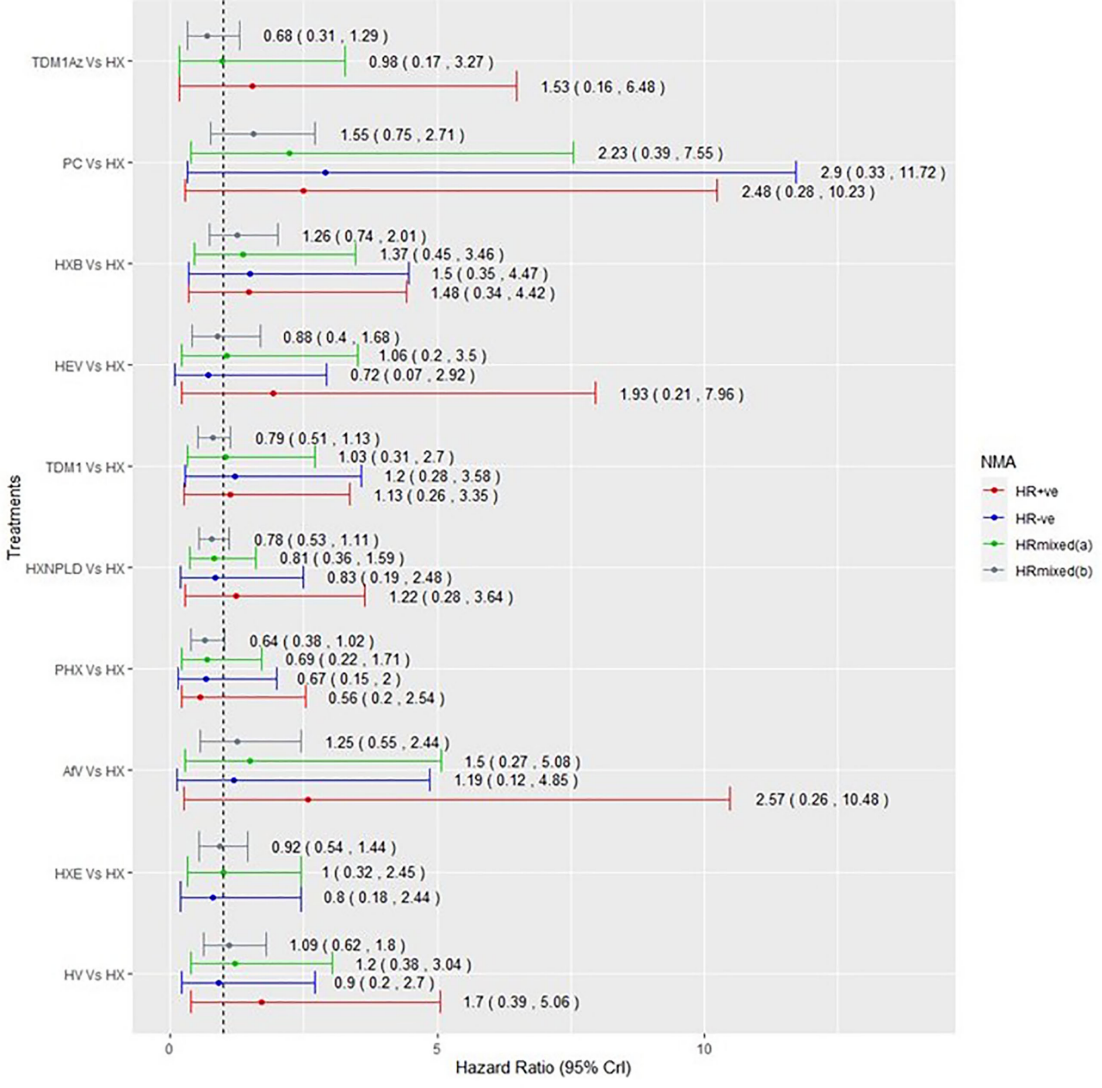
Comparative summary forest plots of treatment effects obtained from the HX connected network for PFS. PC, physician choice; LC, lapatinib + capecitabine; TDM1, trastuzumab emtansine; TDM1AZ, trastuzumab emtansine + atezolizumab; HX, trastuzumab + taxane; HXB, trastuzumab + taxane + bevacizumab; LX, lapatinib + taxane; HV, trastuzumab + vinorelbine; HXE, trastuzumab + taxane + everolimus; PHX, pertuzumab + trastuzumab + taxane; AfV, afatinib + vinorelbine; HEV, trastuzumab + everolimus + vinorelbine; HXNPLD, trastuzumab + taxane + NPLD; NPLD, non-pegylated liposomal doxorubicin.

**Figure 6:**
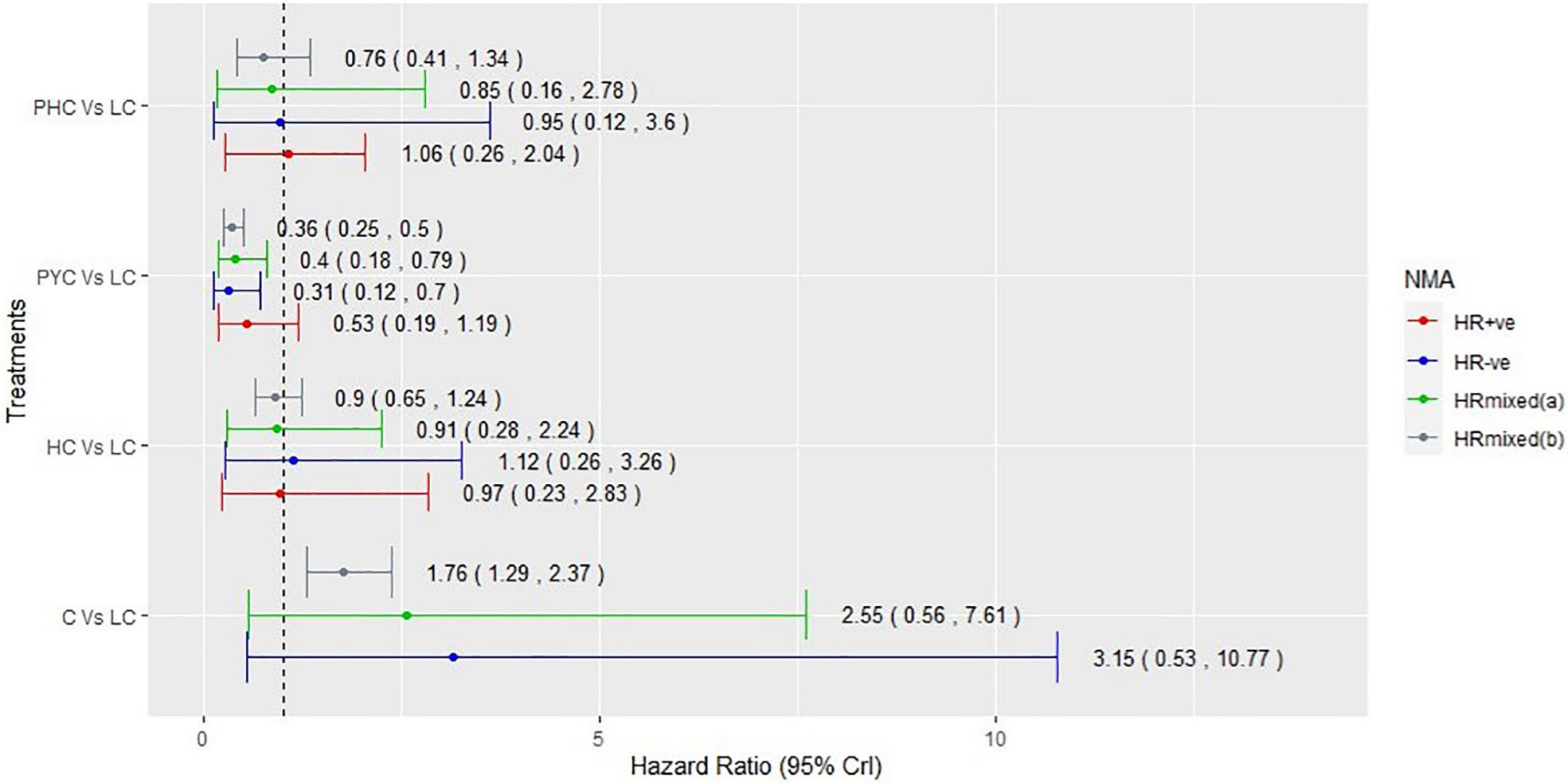
Comparative summary forest plots of treatment effects obtained from capecitabine connected network for PFS. PHC, pertuzumab + trastuzumab + capecitabine; LC, lapatinib + capecitabine; C, capecitabine; PYC, pyrotinib + capecitabine; HC, trastuzumab + capecitabine.

Among the 15 RCTs that recruited the mixed populations of hormone-receptor status patients and reported their subgroup analyses; 13 RCTs reported results for HR+ve patients and 14 RCTs reported results for HR-ve patients. The number of treatment regimens evaluated in the hormone-receptor subgroups (16) was smaller than the treatment regimens evaluated in the overall NMA (26). These do not include treatment regimens in the AI- and HChem-connected network, as RCTs in both connected networks have primarily HR+ve participants. The network plots of RCTs within the hormone-receptor subgroups are displayed in **Figure 4**. The RCTs that reported results for the hormone-receptor subgroups formed two disconnected networks in the subgroup analysis: HX-connected network, and capecitabine-connected network. **Figures 5**, **6** shows the summary forest plots of treatment effects for PFS in the hormone-receptor subgroups, respectively, for the HX-connected network and capecitabine-connected network. The treatment effects from the HR+ve subgroup and HR-ve subgroup are depicted with red and blue bar plots, respectively. The green bar plots show the estimated treatment effects for the mixed patients using only RCTs that reported subgroup analysis, and the gray bar plots depict the treatment effects extracted from the overall NMA including all RCTs (**Figure 3**). In the subgroup analysis, PYC showed a meaningful reduction in the risk of disease progression compared to lapatinib with capecitabine (LC) in the HR-ve subgroup analysis with a hazard ratio of 0.31 (95%Crl: 0.12, 0.70). Other treatment regimens evaluated in the capecitabine- or HX-connected network did not show a meaningful effect as the 95% CrIs included the point of no difference (value of 1).

## Discussion and conclusion

We have conducted the first review of RCTs involving HER2-positive ABC, specifically focusing on the reporting of treatment effects by the hormone receptor status. We found that the RCTs that reported subgroup analyses reported PFS, not OS or ORR. We would like to note that despite PFS being the primary endpoint of these RCTs, the evidence of its surrogacy for OS in HER2-positive ABC is limited (86).

Our results show that, regardless of the hormone-receptor status of the patients, taxane-only therapies were associated with an important increased risk of disease progression compared to HX as well as to other targeted therapies combined with a taxane (as shown in **Supplementary File 3**). This supports the findings from the wider literatures (7, 45, 48, 59, 66). PYC showed a meaningful effect over HX with a hazard ratio of 0.44 (95% Crl: 0.20, 0.82). In the subgroup analyses, PYC showed a meaningful effect over LC in the HR-ve subgroup analysis with a hazard ratio of 0.31 (95% Crl: 0.12, 0.70) and the mixed patients’ analysis with a hazard ratio of 0.40 (95% Crl: 0.18, 0.79).

In addition, our results indicate that the point estimates of HER2 treatments in combination with an AI show a meaningful effect over AI alone, which support the findings by Kawalec et al. (13).

One of the limitations of the review, from the point of view of the clinical interpretation, was the fact that our NMA for both the overall population and the hormone-receptor subgroups included all RCTs that evaluated targeted therapies in HER2-positive patients irrespective of their line of treatments. We chose this approach to capture accumulating relevant evidence available in the reporting of hormone receptor subgroup analysis in the RCTs, as the primary aim of this review was to assess the level of reporting of the effectiveness of therapies in the biomarker subgroups and the impact of under-reporting on the results of NMA. The non-homogeneity of the included RCTs in terms of the treatment line could have played a significant role in the results obtained from the NMA. For example, as mentioned in the Results section, the three RCTs that evaluated PYC in comparison to either LC or capecitabine recruited HER2-positive ABC patients whose disease has progressed after receiving HX, which could have resulted in a meaningful and relatively large treatment difference between PYC and HX. The conclusions drawn from these results are not specific to the line of therapy, and, therefore, the clinical interpretation of these results is limited. Moreover, the sparse and almost-star-shaped geometry of the network as well as the lack of a direct evidence of PYC with other HER2-targeted therapies, such as TDM1, pertuzumab, or HX, mean that there are further limitations of the results in terms of their reliability for the clinical interpretation.

Our review did not identify important differences in treatment effectiveness across hormone-receptor subgroups.

The treatment effect estimates for the subgroup analyses were estimated with increased uncertainty (compared to the mixed population), not only due to the reduced sample size in the subgroups but also due to the limited reporting of the subgroup analyses of the RCTs. However, across treatments, the HR-ve subgroup often presents with a lower estimated hazard ratio than HR+ve patients for PFS. This may therefore warrant a further RCT, powered to investigate the efficacy of HER2-targeted therapies among hormone-receptor subgroups and extending the outcomes assessed by the subgroups to include not only PFS but also OS and ORR. This is because, while PFS may be an attractive primary endpoint as it is available earlier than OS, and is not influenced by subsequent treatments, questions regarding whether PFS is a valid surrogate for OS remain (87–89). Alternatively, an RCT could also be complemented with an analysis of electronic health records (EHRs) to explore if these HER2-targeted therapies are more effective in HR+ve patients compared to HR-ve patients.

Our work serves as an example of exploring the support of a broad evidence base (across treatments) for subgroup effects. It illustrates the evidential and methodological challenges in formally considering subgroup effects using extended networks, which arise due to the limited reporting of subgroup results not only across trials but also across outcomes. This work is still important to inform the value and uncertainty over restricted use in decisions at the national level, such as those facilitated by the NICE in the UK. This is particularly important where the clinical and economic value of a treatment in a particular subgroup is unclear, and therefore, the value of wide adoption is also unclear. In this case, drawing on such an extended evidence base can inform further research recommendations, particularly in considering whether subgroup effects may be generalized across treatments. Our review could be further extended to include data that target the wider HER2 treatment pathway or to include outcomes such as adverse events, the quality or life, or time to progression.

## Author contributions

Conceptualization and design: CMU-C, SB. Data collection: CMU-C, SB. Statistical analysis: CMU-C. Clinical expertise: OA, SK. Manuscript: CMU-C. Critical revision of the manuscript: CMU-C, MS, OA, RO, SK, KA, SB. Supervision: MS, RO, KA, SB. Funding acquisition: SB, KA, RO. All authors contributed to the article and approved the submitted version.

## Funding

This work was supported by Medical Research Council, Methodology Research Panel grant [MR/T025166/1].

## Conflict of interest

SB is a member of the NICE Decision Support Unit. She served as a paid consultant, providing unrelated methodological advice to the NICE, pharmaceutical industry and consultancy companies. She received payments for educational events from Roche and has received research funding from European Federation of Pharmaceutical Industries and Association (EFPAI) and Johnson and Johnson. RO is a member of the National Institute for health and Care Excellence (NICE) Technology Appraisal committee, member of the NICE Decision Support Unit (DSU), and associate member of the NICE Technical Support Unit (TSU). She has served as a paid consultant to the pharmaceutical industry, providing unrelated methodological advice. She reports teaching fees from the Association of British Pharmaceutical Industry (ABPI) and the University of Bristol. KA is a member of the National Institute for Health and Care Excellence (NICE) Diagnostics Advisory Committee and is a National Institute for Health and Care Research (NIHR) Senior Investigator Emeritus. He has acted as a paid consultant, providing unrelated methodological and strategic advice, to the pharmaceutical and life sciences industry generally, as well as to UK Department of Health and Social Care (DHSC)/NICE, and has received unrelated research funding from; Association of the British Pharmaceutical Industry (ABPI), European Federation of Pharmaceutical Industries and Associations (EFPIA), Pfizer, Sanofi and Swiss Precision Diagnostics. He has also received course fees from ABPI and is a Partner/Director of Visible Analytics Limited. SK is supported by a NIHR academic Clinical Lecturer award and has no conflicts of interest to declare. MS is a member of a research funding panel for the National Institute for Health and Care Research (NIHR), and collaborates with the NICE Decision Support Unit (DSU). She has served as a paid consultant to the pharmaceutical and life sciences industry generally, as well as to DHSC/NICE, providing unrelated methodological advice.

The remaining authors declare that the research was conducted in the absence of any commercial or financial relationships that could be construed as a potential conflict of interest.

## Publisher’s note

All claims expressed in this article are solely those of the authors and do not necessarily represent those of their affiliated organizations, or those of the publisher, the editors and the reviewers. Any product that may be evaluated in this article, or claim that may be made by its manufacturer, is not guaranteed or endorsed by the publisher.

## Supplementary material

Supplementary file 1 Name: Searchterms.docx. Description: List of the search terms used in the review

Supplementary file 2 Name: IncludedRCTs.docx. Description: List of RCTs included in the review

Supplementary file 3 Name: PFS treatment effects.xls. Description: Relative treatment effects of the targeted therapies evaluated
