# Supplementary material for: Comparative review of pharmacological therapies in individuals with HER2-positive advanced breast cancer with focus on hormone receptor subgroups
Source: Front Oncol. 2022 Aug 18;12:943154. doi: 10.3389/fonc.2022.943154 (PMC9433866; doi:10.3389/fonc.2022.943154)
Supplement: Supplementary file 1 — Name: Searchterms.docx. Description: List of the search terms used in the review [file DataSheet_1.docx]

**Title:** Comparative review of pharmacological therapies in individuals with HER2 positive advanced breast cancer with focus on hormone receptor subgroup.

Chinyereugo M. Umemneku-Chikere^1^, Olubukola Ayodele^2^, Marta Soares^3^, Sam Khan^4^, Keith Abrams^5^, Rhiannon Owen^6^, Sylwia Bujkiewicz^1^

^1^Biostatistics Research Group, Department of Health Sciences, University of Leicester, Leicester, UK

^2^University Hospital Leicester NHS Trust, Leicester Royal Infirmary, Leicester, UK

^3^Centre for Health Economics, University of York, UK

^4^Leicester Cancer Research Centre, Robert Kilpatrick Clinical Sciences Building, University of Leicester, Leicester, UK

^5^Department of Statistics, University of Warwick, UK

^6^Swansea University, Medical School, Swansea University, UK

Email: [cmuc1@leicester.ac.uk](mailto:cmuc1@leicester.ac.uk)

**Search term**:

The search terms include: **(((HER2 positive) AND ("metastatic breast cancer" OR "MBC" OR "advanced breast cancer" OR "ABC" OR "stage IV")) AND ("female" OR "women" OR "woman")) AND ("NMA" OR "systematic review" OR "meta-analysis" OR "network meta-analysis").**
