# Supplementary material for: Comparative review of pharmacological therapies in individuals with HER2-positive advanced breast cancer with focus on hormone receptor subgroups
Source: Front Oncol. 2022 Aug 18;12:943154. doi: 10.3389/fonc.2022.943154 (PMC9433866; doi:10.3389/fonc.2022.943154)
Supplement: Supplementary file 2 — Name: IncludedRCTs.docx. Description: List of RCTs included in the review [file DataSheet_2.docx]

**Title:** Comparative review of pharmacological therapies in individuals with HER2 positive advanced breast cancer with focus on hormone receptor subgroup.

Chinyereugo M. Umemneku-Chikere^1^, Olubukola Ayodele^2^, Marta Soares^3^, Sam Khan^4^, Keith Abrams^5^, Rhiannon Owen^6^, Sylwia Bujkiewicz^1^

^1^Biostatistics Research Group, Department of Health Sciences, University of Leicester, Leicester, UK

^2^University Hospital Leicester NHS Trust, Leicester Royal Infirmary, Leicester, UK

^3^Centre for Health Economics, University of York, UK

^4^Leicester Cancer Research Centre, Robert Kilpatrick Clinical Sciences Building, University of Leicester, Leicester, UK

^5^Department of Statistics, University of Warwick, UK

^6^Swansea University, Medical School, Swansea University, UK

Email: [cmuc1@leicester.ac.uk](mailto:cmuc1@leicester.ac.uk)

Table 1: List of RCTs included in this review

| **Author(s)** | **Year** | **Treatments** | **Number of patients** | **HR Status** | **Outcomes measured** |
| --- | --- | --- | --- | --- | --- |
| **Aromatase Inhibitor connected RCTs** | | | | | |
| Johnston et al | 2009 | LAI (E) | 111 | Positive | PFS, OS, ORR |
|  |  | AI (C) | 108 |  |  |
| Kaufman et al | 2009 | HAI (E) | 103 | Positive | PFS, ORR |
|  |  | AI (C) | 104 |  |  |
| Mehta et al | 2012 | FAI (E) | 56 | Positive | PFS |
|  |  | AI (C) | 31 |  |  |
| Huober et al | 2012 | HAI (E) | 26 | Positive | PFS, ORR |
|  |  | AI (C) | 31 |  |  |
| Rimawi et al | 2018 | PHAI (E) | 129 | Positive | PFS, ORR |
|  |  | AI (C) | 129 |  |  |
| **Trastuzumab – Taxane connected RCTs** | | | | | |
| Andersson et al | 2011 | HX (E) | 143 | Mixed | PFS, OS, ORR |
|  |  | HV (C) | 141 |  |  |
| Andre et al | 2014 | HEV (E) | 284 | Mixed | PFS, ORR |
|  |  | HV (C) | 285 |  |  |
| Awada et al | 2016 | NX (E) | 242 | Mixed | PFS, OS, ORR |
|  |  | HX (C) | 237 |  |  |
| Baselga et al | 2012 | PHX (E) | 402 | Mixed | PFS, OS, ORR |
|  |  | HX (C) | 404 |  |  |
| Baselga et al | 2014 | HXNPLD (E) | 181 | Mixed | PFS, OS |
|  |  | HX (C) | 182 |  |  |
| Burstein et al | 2007 | HV (E) | 41 | Mixed | ORR |
|  |  | HX (C) | 40 |  |  |
| Cameron et al | 2010 | LC (E) | 198 | Mixed | PFS, OS, ORR |
|  |  | C (C) | 201 |  |  |
| Di Leo et el | 2008 | LX (E) | 49 | Mixed | PFS, OS, ORR |
|  |  | X (C) | 37 |  |  |
| Emens et al | 2020 | TDM1AZ (E) | 133 | Mixed | PFS, OS, ORR |
|  |  | TDM1 (C) | 69 |  |  |
| Gasparini et al | 2009 | HX (E) | 63 | Mixed | PFS, ORR |
|  |  | X (C) | 60 |  |  |
| Gelmon et al | 2015 | LX (E) | 326 | Mixed | PFS, OS |
|  |  | HX (C) | 326 |  |  |
| Geyer et al | 2006 | LC (E) | 163 | Mixed | PFS, OS, ORR |
|  |  | C (C) | 161 |  |  |
| Gianni et al | 2013 | HX (E) | 206 | Mixed | PFS, ORR |
|  |  | HXB (C) | 215 |  |  |
| Guan et al | 2013 | LX (E) | 222 | Mixed | PFS, OS, ORR |
|  |  | X (C) | 222 |  |  |
| Harbeck et al | 2016 | AfV (E) | 339 | Mixed | PFS, OS, ORR |
|  |  | HV (C) | 169 |  |  |
| Hurvitz et al | 2015 | HXE (E) | 480 | Mixed | PFS, ORR |
|  |  | HX (C) | 239 |  |  |
| Hurvitz et al | 2013 | TDM1 (E) | 67 | Mixed | PFS, ORR |
|  |  | HX (C) | 70 |  |  |
| Jianni et al | 2014 | LV | 75 | Mixed | PFS, OS, ORR |
|  |  | LC | 37 |  |  |
| Krop et al | 2014 | TDM1 | 404 | Mixed | PFS, OS |
|  |  | PC | 198 |  |  |
| Ma et al | 2019 | PYC | 65 | Mixed | PFS |
|  |  | LC | 63 |  |  |
| Martin et al | 2013 | N | 116 | Mixed | PFS, OS, ORR |
|  |  | LC | 116 |  |  |
| Marty et al | 2005 | HX | 92 | Mixed | PFS, OS, ORR |
|  |  | X | 94 |  |  |
| Perez et al | 2017 | PTDM1 | 363 | Mixed | PFS, OS, ORR |
|  |  | HX | 365 |  |  |
|  |  | TDM1 | 367 |  |  |
| Pivot et al | 2015 | LC | 271 | Mixed | PFS, OS, ORR |
|  |  | HC | 269 |  |  |
| Robert et al | 2006 | HXCb | 98 | Mixed | PFS, OS, ORR |
|  |  | HX | 98 |  |  |
| Saura et al | 2020 | NC | 307 | Mixed | PFS, OS, ORR |
|  |  | LC | 314 |  |  |
| Slamon et al | 2001 | HX | 92 | Mixed | PFS, OS, ORR |
|  |  | X | 96 |  |  |
| Tanko et al | 2018 | HC | 43 | Mixed | PFS, OS, ORR |
|  |  | LC | 43 |  |  |
| Urruticoechea et al | 2016 | PHC | 228 | Mixed | PFS, OS, ORR |
|  |  | HC | 224 |  |  |
| Valero et al | 2011 | HX | 131 | Mixed | PFS, OS, ORR |
|  |  | HXCb | 132 |  |  |
| Verma et al | 2012 | TDM1 | 495 | Mixed | PFS, OS, ORR |
|  |  | LC | 496 |  |  |
| Von Minckwitz et al | 2009 | HC | 78 | Mixed | PFS, OS, ORR |
|  |  | C | 78 |  |  |
| Wardley et al | 2010 | HXC | 112 | Mixed | PFS, ORR |
|  |  | HX | 110 |  |  |
| Xu et al | 2021 | PYC | 134 | Mixed | PFS, ORR |
|  |  | LC | 132 |  |  |
| Yan et al | 2020 | PYC | 185 | Mixed | PFS, ORR |
|  |  | C | 94 |  |  |
| **Chemotherapy connected RCTs** | | | | | |
| Salmon et al | 2001 | HChemo (E) | 235 | Mixed | PFS, OS, ORR |
|  |  | Chemo (C) | 234 |  |  |
| Tolaney et al | 2020 | HAb (E) | 79 | Positive | PFS, ORR |
|  |  | HChemo (C) | 79 |  |  |
| Tolaney et al | 2020 | HAbF (E) | 79 | Positive | PFS ORR |
|  |  | HChemo (C) | 79 |  |  |
| **Others** | | | | | |
| Blackwell et al | 2012 | LH (E) | 145 | Mixed | PFS, OS, ORR |
|  |  | L (C) | 146 |  |  |
| **Abbreviations**: *PHC* pertuzumab + trastuzumab + capecitabine; *PC* physician choice; *LC* lapatinib + capecitabine; *TDM1* trastuzumab emtansine; *C* capecitabine; *PYC* pyrotinib + capecitabine; *LV* lapatinib + vinorelbine; *HC* trastuzumab + capecitabine; *N* is neratinib; *TDM1AZ* trastuzumab emtansine + atezolizumab; *NX* neratinib + taxane; *X* taxane (paclitaxel or docetaxel);*NC* neratinib + capecitabine; *HX* trastuzumab + taxane; *HXB* trastuzumab + taxane + bevacizumab; *LX* lapatinib + taxane; *HV* trastuzumab + vinorelbine; *HXE* trastuzumab + taxane + everolimus; *PHX* pertuzumab + trastuzumab + taxane; *HXC* trastuzumab + capecitabine + taxane; *AfV* is afatinib + vinorelbine; *HEV* is trastuzumab + everolimus + vinorelbine; *HXCb* trastuzumab + taxane + carboplatin; *PTDM1* pertuzumab + trastuzumab emtansine; *Chemo* standard chemotherapy; *LH* lapatinib + trastuzumab; *L* lapatinib; *AI* aromatase inhibitors (letrozole or anastrozole); *LAI* lapatinib + AI; *FAI* fulverstrant + AI; *HAI* trastuzumab +AI; *PHAI* pertuzumab + trastuzumab +AI; *HAb* trastuzumab + abemaciclib; *HAbF* trastuzumab + abemaciclib + fulverstrant; *HXNPLD* trastuzumab + taxane + NPLD; *NPLD* non-pegylated liposomal doxorubicin; *HChemo* trastuzumab + chemotherapy, *Eb* eribulin. | | | | | |
